# Data‐Driven Cycle Life Prediction of Lithium Metal‐Based Rechargeable Battery Based on Discharge/Charge Capacity and Relaxation Features

**DOI:** 10.1002/advs.202402608

**Published:** 2024-06-27

**Authors:** Qianli Si, Shoichi Matsuda, Youhei Yamaji, Toshiyuki Momma, Yoshitaka Tateyama

**Affiliations:** ^1^ Department of Nanoscience and Nanoengineering Faculty of Science and Engineering Waseda University 3‐4‐1 Okubo Shinjuku‐ku 169‐8555 Japan; ^2^ Research Center for Energy and Environmental Materials (GREEN) National Institute for Materials Science (NIMS) 1‐1 Namiki Tsukuba Ibaraki 305‐0044 Japan; ^3^ NIMS‐SoftBank Advanced Technologies Development Center National Institute for Materials Science (NIMS) 1‐1 Namiki Tsukuba Ibaraki 305‐0044 Japan; ^4^ Laboratory for Chemistry and Life Science Institute of Innovative Research Tokyo Institute of Technology 4259 Nagatsuta‐cho Midori‐ku Yokohama 226‐8501 Japan

**Keywords:** battery, cycle life, features, Li metal anode, machine learning model

## Abstract

Achieving precise estimates of battery cycle life is a formidable challenge due to the nonlinear nature of battery degradation. This study explores an approach using machine learning (ML) methods to predict the cycle life of lithium‐metal‐based rechargeable batteries with high mass loading LiNi_0.8_Mn_0.1_Co_0.1_O_2_ electrode, which exhibits more complicated and electrochemical profile during battery operating conditions than typically studied LiFePO₄/graphite based rechargeable batteries. Extracting diverse features from discharge, charge, and relaxation processes, the intricacies of cell behavior without relying on specific degradation mechanisms are navigated. The best‐performing ML model, after feature selection, achieves an *R* ^2^ of 0.89, showcasing the application of ML in accurately forecasting cycle life. Feature importance analysis unveils the logarithm of the minimum value of discharge capacity difference between 100 and 10 cycle (Log(|min(Δ*DQ*_ 100–10_(V))|)) as the most important feature. Despite the inherent challenges, this model demonstrates a remarkable 6.6% test error on unseen data, underscoring its robustness and potential for transformative advancements in battery management systems. This study contributes to the successful application of ML in the realm of cycle life prediction for lithium‐metal‐based rechargeable batteries with practically high energy density design.

## Graphical abstract

Discover the potential of machine learning in predicting LiNi_0.8_Mn_0.1_Co_0.1_O_2_ Li metal battery (NMC811/LMB) cycle life. In‐house NMC811/LMB degradation datasets are generated, through feature selection and optimization, XGBoost is selected for superior prediction accuracy, and the best ML model achieves an impressive *R* ^2^ = 0.89. Insights are offered into the future of battery degradation analysis and energy storage.

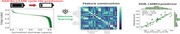

## 1 Introduction

Lithium‐ion batteries (LIBs) are extensively utilized as energy storage tools in various industries such as electric vehicles, portable electronic devices, and grid energy because of their remarkable properties such as high energy density, low self‐discharging rate, affordability, and prolonged lifespan.^[^ ^1^, ^2^, ^3^ ^]^ Nevertheless, like numerous other electrochemical systems, LIBs experience unavoidable energy and power degradation over time, leading to diminished capacity and increased internal resistance.^[^ ^4^ ^]^ Therefore, precisely predicting the cycle life of LIBs can help industries optimize battery usage, replacement schedules, reducing unnecessary replacements and associated costs. In addition, researchers can evaluate the quality of batteries in advance which enables them to identify potential issues and optimize battery design.^[^ ^5^, ^6^ ^]^

Due to the complex degradation mechanisms and non‐linear degradation patterns of LIBs, predicting their lifetime is challenging. In previous research, the strategies for battery lifetime prediction are classified into three main groups: mechanism methods,^[^ ^7^, ^8^ ^]^ model‐based methods,^[^ ^9^, ^10^, ^11^ ^]^ and data‐driven methods.^[^ ^12^, ^13^ ^]^ Among them, data‐driven methods that use statistical data and machine learning (ML) algorithms have recently attracted great attention because of the big data era.

In recent year, there have been several reports on predicting the cycle life of commercial LIBs which are already quite mature and stable.^[^ ^14^, ^15^ ^]^ Wu et al. demonstrated the feasibility of online remaining useful life (RUL) estimation using a feed‐forward neural network. Their study achieved an error of less than 5% in predicting the cycle life within the practical operation, though it's based on the data of only two LIBs.^[^ ^16^ ^]^ Severson et al. conducted a study where they examined 124 rechargeable batteries under fast charging conditions in an experiment. These batteries were commercial lithium iron phosphate/graphite cells and were maintained at a forced convection temperature of 30 °C throughout the tests. Various parameters of the discharge process were monitored during the experiments. Through the experimental data, combined with the ML algorithm (ElasticNet), the cycle life of the 124 batteries was predicted successfully with a 9.1% test error compared with the observed cycle life.^[^ ^17^ ^]^ This work is regarded as the pioneer of this research field since the dataset used in this study is the largest publicly available for nominally identical commercial LIBs that were cycled under controlled conditions. Inspired by this, various research groups have attempted to employ different ML models and features to predict the cycle life of commercial LIBs.^[^ ^18^, ^19^ ^]^ The use of various battery cell systems and the application of different ML methods display the effectiveness of cycle life prediction.

Lithium metal‐based rechargeable battery (LMB) have attracted much attention for their high specific capacity (3860 mAh/g) that allows for the lowest electrochemical potential (−3.04 V vs the standard hydrogen electrode) and energy density, which extends range for electric vehicles, and improved performance in various energy‐intensive applications.^[^ ^20^, ^21^ ^]^ Actually, by combining the high‐capacity Ni‐rich LiNi_0.8_Mn_0.1_Co_0.1_O_2_ (NMC) electrode, LMB with cell level energy density over 350 Wh kg^−1^ has been reported for realizing stable charge/discharge reaction more than 200 cycles.^[^ ^22^ ^]^ However, compared to the conventional graphite‐based LIBs, LMB has lower redox potential leading to easy reductive decomposition of electrolytes, resulting in complicated degradation reaction of lithium metal electrodes. For example, the dendritic growth of metallic lithium is widely recognized as a crucial problem of lithium metal electrodes, where needle‐like structures form on the surface of the electrode during cycling that can lead to short circuits and battery failure.^[^ ^20^ ^]^

There have been some studies to model the aging of LMB so far. Gao et al. devised a method for real‐time detection of LMB failure modes by monitoring changes in rest voltage and Coulombic efficiency. The study also introduced an accelerated lifetime testing method to predict the maximum lifetime of LMBs based on a dominant ultimate failure mechanism.^[^ ^23^ ^]^ Dessantis et al. developed a pseudo‐2D aging electrochemical model for a lithium metal–LiFePO_4_L battery, accurately representing its electrochemical behavior across different charge rates and predicting discharge capacity loss for multiple cycles.^[^ ^24^ ^]^ However, most of the previous works relied on mechanistic or model‐based methods, requiring significant computational resources for detailed analysis and lacking sufficient validation. Furthermore, recent intensive investigations utilizing various analytical techniques have unveiled the formation of isolated metallic lithium during repeated cycling, leading to substantial volume expansion of the lithium metal electrode.^[^ ^25^ ^]^ Besides, internal resistance significantly increases due to the electrolyte shortage,^[^ ^26^ ^]^ and, in practical cell design conditions, chemical crossover reactions between electrodes must be considered. In summary, the diverse degradation mechanisms and unique challenges posed by safety concerns as well as the limited data availability of LMB highlight the need for tailored ML‐based cycle life prediction methods. The ML approaches may offer more flexibility and mechanism‐free characteristics compared to traditional model‐based or mechanistic methods, making them well‐suited to address the complexities inherent in LMB aging prediction.

Under such circumstances, we addressed the construction of an ML model for high cell‐level energy density LMBs. In the present study, 57 cells of 350 Wh/kg^cell^ class LMB were fabricated using high mass loaded Ni‐rich NMC electrode, 48 out of the 57 cells were used to construct the ML model, the remaining nine cells were regarded as unseen data. 35 features were generated from raw cell data of the first 100 cycles, which were classified into three groups: charge, relaxation, and discharge‐related features. Initially, the linear regression model ElasticNet^[^ ^27^ ^]^ was applied to the three feature subsets independently to predict the cycle life of the cells. Regrettably, the predictive performance of the model did not meet the desired standards. Then, the correlation of the 35 features to the observed cycle life was systematically studied by calculating Pearson's correlation coefficient. 12 features that exhibit strong or moderate correlations to the cycle life were extracted. Based on these 12 features, combining exhaustive feature selection, the prediction performance of non‐linear regression model XGBoost^[^ ^28^ ^]^ was analyzed. We then found that using XGBoost and the selected feature subset which contains six features showed the best prediction result with *R* ^2^ = 0.89 and a Root mean square error (RMSE) of 8.29. Finally, we applied our ML model to eight unseen cells (Though nine cells were prepared originally, 1 cell was eventually excluded because of the unstable capacity profile). The best test error of the cycle life is 6.6%.

## 2 Computational Section

### 2.1 LMB Fabrication and Performance Test

**Figure 1:**
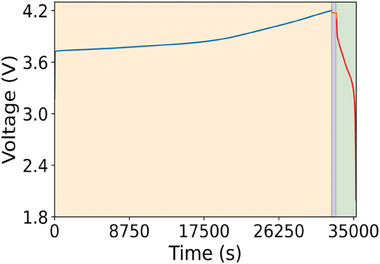
The cycling data for one representative cell in the first cycle. The charging phase is denoted by the yellow segment, followed by a relaxation period indicated in blue. Subsequently, the discharging phase is represented by the green section.

In our project, total 57 monolayer stacked pouch‐type LMB cells (48 cells for model construction, 9 cells as unseen data) were assembled, consisting of a positive electrode made of NMC811 (40 mm x 30 mm) with mass loading of 30 mg cm^−2^, a separator (6 mm × 36 mm), and a negative electrode comprising a 50 µm thick layer of lithium on a 10 µm thick copper (Cu) current collector (42 mm × 32 mm). The details of electrolyte solution and separator used in the present study are summarized in the supporting information (Table S1, Supporting Information). All the cells were fabricated inside a dry room (dew point < −50 °C) and electrolyte injection was carried out inside a fume hood (dew point < −85 °C). The details of the charge/discharge test conditions are also described in supporting information. Charge and discharge of the cells were carried out with Hokuto Denko HJ1001SD8 at 25 °C. All the cells were cycled at constant current in the voltage range 2–4.2 V. Voltage, current, and capacity of the cells were continuously recorded during the cycling process, through this process, we obtained the charge‐discharge curves at all the cycles as well as the discharge capacity retention curve. One complete cycling curve of one specific cell is shown in **Figure** **1**, which includes 3 processes 1) constant‐current charging, 2) relaxation after charge, 3) constant‐cCC discharging.

### 2.2 Feature Construction

In the present study, a total of 35 features were extracted from the raw voltage and capacity data obtained during the entire cycling test, encompassing charge, discharge, and voltage relaxation processes. These features were systematically categorized into three distinct groups: discharge‐related features, charge‐related features, and relaxation‐related features. While previous research predominantly focused on features generated solely from the discharge process, with minimal attention given to those derived from voltage relaxation or charge processes, our study sought to bridge this gap by incorporating features from all three processes concurrently to assess the model's performance. Here, we not only generated features from the discharge process that have been typically used in previous work,^[^ ^17^ ^]^ but also calculated capacity retention of different cycles. Additionally, we introduced novel features derived from both charge and relaxation processes within the initial 100 cycles. This strategy aimed to determine the relative importance of each process in predicting cycle life while simultaneously enhancing prediction performance by selecting features from all three processes. Furthermore, it facilitated the selection of features from diverse processes, thereby improving the overall predictive capabilities of the model.

#### 2.2.1 Discharge‐Related Features

Seventeen features were derived from the discharge process, among which six features were calculated as summary statistics: minimum, variance, skewness, kurtosis, mean, and the initial value of the change in discharge voltage curves between different cycles *(ΔDQ *(V)) extracted from the discharge capacity‐voltage curves. These curves capture the electrochemical evolution of individual cells during cycling, thereby encoding valuable insights into cell degradation mechanisms. Summary statistics were demonstrated to effectively elucidate the shape and positional changes of the voltage curve, offering a succinct representation of its characteristics. In the present study, we choose these summary statistics based on their predictive power rather than their direct physical significance.

**Figure 2:**
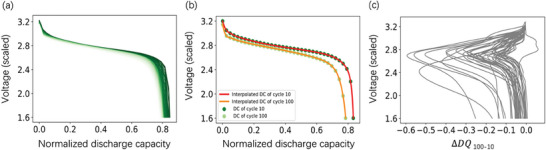
a) Discharge profile from 1st cycle to 100th cycle of cell No. 32 in our dataset. The color changed from dark to light as the cycle number increased. b) Discharge capacity ‐Voltage curves for 10th and 100th cycles for cell No. 32. The abbreviation “DC” denotes discharge capacity. c) Discharge capacity difference (Δ*DQ*_ 100–10_) as a function of voltage between the 10th and 100th cycles for 40 cells.

Δ*DQ* (V) serves as a pivotal metric, quantifying the disparity between discharge capacity‐voltage curves of two cycles. Specifically, Δ*DQ*_ 100–10_(V) is computed as the difference between the discharge capacities at the 100th cycle and the 10th cycle, denoted as DQ_100_(V) – DQ_10_(V), employing interpolated discharge capacity data. This metric highlights the variation in discharge capacity‐voltage curves between the 100th and 10th cycles, providing crucial insights into cell degradation dynamics. In **Figure** **2**a, we present the original discharge profiles spanning from the 1st cycle to the 100th cycle of cell No. 32 within our dataset. To visualize degradation, discharge capacity‐voltage curves corresponding to the 10th cycle and 100 of this cell are extracted and depicted in Figure 2b. Subsequently, in Figure 2c, the Δ*DQ*_ 100–10_(V) curves for all 40 cells are illustrated. In an ideal scenario, the difference in the discharge capacity curves between the 100th and 10th cycles would typically exhibit negative values, indicating a decrease in discharge capacity over time. However, our analysis revealed instance where one cell displayed positive segments in this curve, contrary to the expected trend. We attribute this unexpected observation to phenomena such as electrode activation or capacity recovery. Notably, as part of our data processing and visualization methodology, we uniformly scaled the voltage values by a factor of 0.8 for enhanced presentation clarity. This scaling adjustment was uniformly applied across all voltage values in the dataset.

In addition, the discharge capacity of the 2nd cycle, the 10th cycle, and the 100th cycle were extracted, which quantifies the energy output of the cell within a cycle and how it changes over time. Moreover, by using the discharge capacity of the 2nd, the 10th and the 100th cycle, we calculated the capacity retention (CR), a fundamental metric of the discharge capacity at different cycle, which serves as a crucial metric for accessing cell degradation. Which is defined as the ratio of discharge capacity at cycle n *C_Dch_* (n) to that of cycle n −1 *C_Dch_* (n‐1)

(1) $$CR\;=\frac{C_{Dch\left(n\right)}}{C_{Dch\left(n-1\right)}}$$

Then we calculated features such as the slope and the intercept of the discharge curve between the 2nd cycle the 100th and those of the 91st cycle to the 100th cycle.

#### 2.2.2 Charge‐Related Features

From the charging process, we generated 12 features, including six summary statistics: minimum, variance, skewness, kurtosis, mean, and the initial value of the change in charge voltage curves between different cycles (Δ*CQ*(V)) extracted from the charge capacity‐voltage curves. These features exhibit crucial information regarding the charging behavior of the cells and offer valuable insights into their performance characteristics.

**Figure 3:**
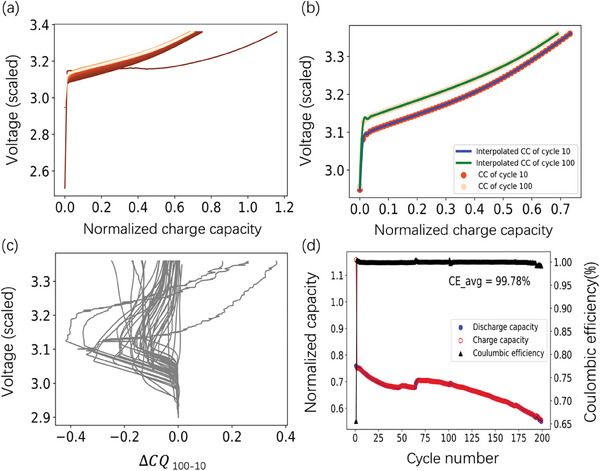
a) Charge profile from the 1st cycle to the 100th cycle of cell No. 41 in our dataset. The color gradient shifts from dark to light as the cycle number increases. b) Charge capacity‐voltage curves for the 10th and 100th cycles of cell No. 41. The abbreviation “CC” denotes charge capacity c) Δ*CQ*_ 100–10_(V) as a function of voltage between the 10th and 100th cycles for 40 cells. d) Discharge capacity, charge capacity, and coulombic efficiency as a function of the cycle number of cell No. 41.

In **Figure** **3**a, we present the original charge profile spanning from the 1st cycle to the 100th cycle of cell No. 41 within our dataset. To provide further insight into the charging behavior, charge capacity–voltage curves corresponding to the 10th cycle to the 100th cycle of this cell are extracted and depicted in Figure 3b. Additionally, Figure 3c illustrates the Δ*CQ*_ 100–10_(V) curves for all 40 cells in our dataset, offering a comprehensive view of the charging dynamics across the sampled cells. Similar to the discharge process, three cells’ Δ*CQ*_ 100–10_(V) curves showed positive segments in the curve.

Charge capacity of the 2nd, the 10th, and the 100th cycle were also included. Coulombic efficiencies (CEs) of those cycles calculated from charge capacity of the 2nd, the 10th, and the 100th cycle, respectively, were also selected as features to predict the cell cycle life. The CE of cycle *n* is defined as the ratio of measured discharge capacity of cycle n *C_Dch_* (*n*) and measured the charge capacity of cycle n *C_Ch_* (n)

(2) $$CE\;=\frac{C_{Dch\left(n\right)}}{C_{Ch\left(n\right)}}$$

Typically, for an idealized cell where side‐reactions are absent, Coulombic Efficiency (CE) reaches unity due to the absence of losses in both lithium transfer and electron transfer processes. The Coulombic Efficiency of the cells used in our project were all recorded, which is displayed in Figure 3d. By tracking these features across multiple cycles, we gain insights into how the cell's performance changes over time and how it evolves through repeated charge‐discharge cycles. In addition, these features represent different stages of cell operation, including initial conditioning, mid‐term performance, and long‐term degradation. By including them, we capture a comprehensive view of the cell's behavior across its operational lifespan, which may enhance the predictive capabilities of the model.

#### 2.2.3 Relaxation Related Features

**Figure 4:**
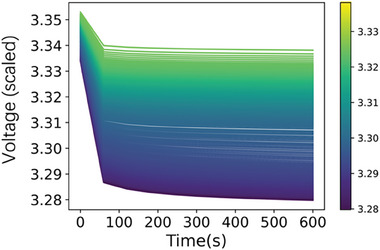
Relaxation voltage as a function of the relax time of cell No. 13 from the first cycle to the last cycle (200 cycles). The color of the line is differentiated by the voltage at 600s.

It has been demonstrated that the relaxation process, encompassing the voltage value during a particular time interval and the voltage curve within a designated timeframe, exhibits a correlation with the State of Health (SoH) of the cell.^[^ ^29^, ^30^, ^31^ ^]^ Six features were generated during the relaxation process, including minimum, maximum, variance, skewness, kurtosis, and mean of the terminal voltage from the 1st cycle to the 100th cycle from the relaxation voltage‐time curves, which provide valuable information about the voltage distribution and dynamics during relaxation periods. These metrics can help in detecting abnormalities or irregularities in the cell's behavior, which may indicate potential degradation mechanisms or performance issues. The voltage of cell No. 13 between the 1st cycle to the 100th cycle during the cell relaxation is shown in **Figure** **4**.

We summarized the total 35 features in **Table** **1**.

**Table 1:** 35 features extracted from discharge, charge, and voltage relaxation process.

Feature types	Feature description
Discharge‐related features	Log(|min(Δ*DQ*_ 100–10_(V))|),Log(|mean(Δ*DQ*_ 100–10_(V))|), Log(|var(Δ*DQ*_ 100–10_(V))|),Log(|Δ*DQ*_ 100–10_(V) [0]|), Log(|skew(Δ*DQ*_ 100–10_(V))|), Log(|Kur(Δ*DQ*_ 100–10_(V))|); Slope and Intercept of the linear fit to the capacity fade curve, cycles 2 to 100, 91 to 100 (Slope_DQ, Intercept_DQ); Discharge capacity of 2, 10, and 100 (DQ_n); Max difference of discharge capacity of 100 and 2; Capacity_retention_1:100, 2:1, 99:100 (CR_n).
Charge‐related features	Log(|min(Δ*CQ*_ 100–10_(V))|),Log(|mean(Δ*CQ*_ 100–10_(V))|), Log(|var(Δ*CQ*_ 100–10_(V))|),Log(|Δ*CQ*_ 100–10_(V) [0]|), Log(|skew(Δ*CQ*_ 100–10_(V))|), Log(|Kur(Δ*CQ*_ 100–10_(V))|); Charge capacity of 2, 10, and 100 (CQ_n); Coulombic efficiency of 2, 10, and 100 (CEn).
Relaxation‐related features	Max, Min, Var, Mean, Skew and Kurtosis for the terminal voltage between 1 and 100 cycles.

### 2.3 Machine Learning Process

The entire dataset was utilized as the training dataset, employing a four‐fold cross‐validation strategy for model evaluation. This methodology involved partitioning the dataset into four mutually exclusive subsets. Iteratively, the model was trained and evaluated four times, with each subset serving as the test set once while the remaining three subsets collectively constituted the training set. By adopting this four‐fold cross‐validation approach, we ensured a thorough and reliable assessment of the model's performance, enhancing our understanding of its generalization capabilities.

In the present study, we employed two machine learning methods: ElasticNet, a linear regression model combining Lasso (L1) and Ridge (L2) regularization techniques, and XGBoost, a nonlinear regression model. ElasticNet was chosen for its ability to handle high‐dimensional datasets with potentially correlated features. By incorporating both L1 and L2 penalties, ElasticNet allows for feature selection and regularization, which can mitigate overfitting and improve generalization performance. XGBoost, on the other hand, is a powerful gradient‐boosting algorithm known for its scalability, efficiency, and ability to handle complex nonlinear relationships in the data. It can capture intricate patterns and interactions in the dataset, leading to enhanced predictive accuracy. Additionally, XGBoost provides insights into feature importance, aiding in the identification of the most relevant variables for prediction. The adoption of XGBoost was motivated by its superior performance in achieving accurate and reliable predictions when ElasticNet failed to produce satisfactory results. Both ElasticNet and XGBoost algorithms are further illustrated in the supporting information.

The performance of the ML models was evaluated by the following three statistical metrics:

Mean absolute error (MAE):

(3) $$MAE\;=\frac{1}{n}\;\sum_{i\;=\;1}^{n}\left|y_{i}-{\hat{y}}_{i}\right|$$

Root mean square error (RMSE):

(4) $$RMSE\;=\sqrt{\frac{1}{n}\sum_{i=1}^{n}\;{\left(y_{i}-{\hat{y}}_{i}\right)}^{2}\;}$$

R^2^:

(5) $$R^{2}=\;1-\frac{\sum_{k\;=\;1}^{n}\;{\left(y_{i}-{\hat{y}}_{i}\right)}^{2}}{\sum_{k\;=\;1}^{n}\;{\left(y_{i}-\bar{y}\right)}^{2}}$$

Here, n represents the number of cells, $y_{i}$ and ${\hat{y}}_{i}$ represents the observed cycle life and the predicted cycle life for sample i. The Mean Absolute Error (MAE) gauges the proximity of predictions to the respective outcomes. On the other hand, the Root Mean Square Error (RMSE), which captures the dispersion of errors, exhibits higher sensitivity toward substantial deviations compared to the MAE. For these two values, small values represent good performance of the models. The *R* ^2^ is a metric expressed in percentages, and in an optimal scenario, *R* ^2^ approaches 100% or 1, indicating a strong alignment between the observed and predicted values.

Furthermore, in our project, we used Pearson's correlation coefficient to depict the relationship between the features and cell cycle life. The coefficient is defined are calculated by^[^ ^32^ ^]^:

(6) $$r\;=\frac{\sum_{i=1}^{p}\left(x_{i}-\bar{x}\right)\left(y_{i}-\bar{y}\right)}{\sqrt{{\left((x_{i}-\bar{x}\right)}^{2}}\;\sqrt{{\left((y_{i}-\bar{y}\right)}^{2}}}$$

The correlation coefficient is bounded within the range of −1 to 1, which signifies the strength and direction of the correlation between two variables, x and y. A positive correlation yields values between 0 and 1, indicating that as one variable increases, the other tends to increase. Conversely, a negative correlation results in values between −1 and 0, implying that as one variable increases, the other tends to decrease. Interpretation of the coefficient involves magnitude: a value exceeding |0.8| points to a strong correlation, while a value lower than |0.5| signifies a weak correlation. Within the range of |0.5| and |0.8|, a correlation of moderate strength is observed.

## 3 Results and Discussion

**Figure 5:**
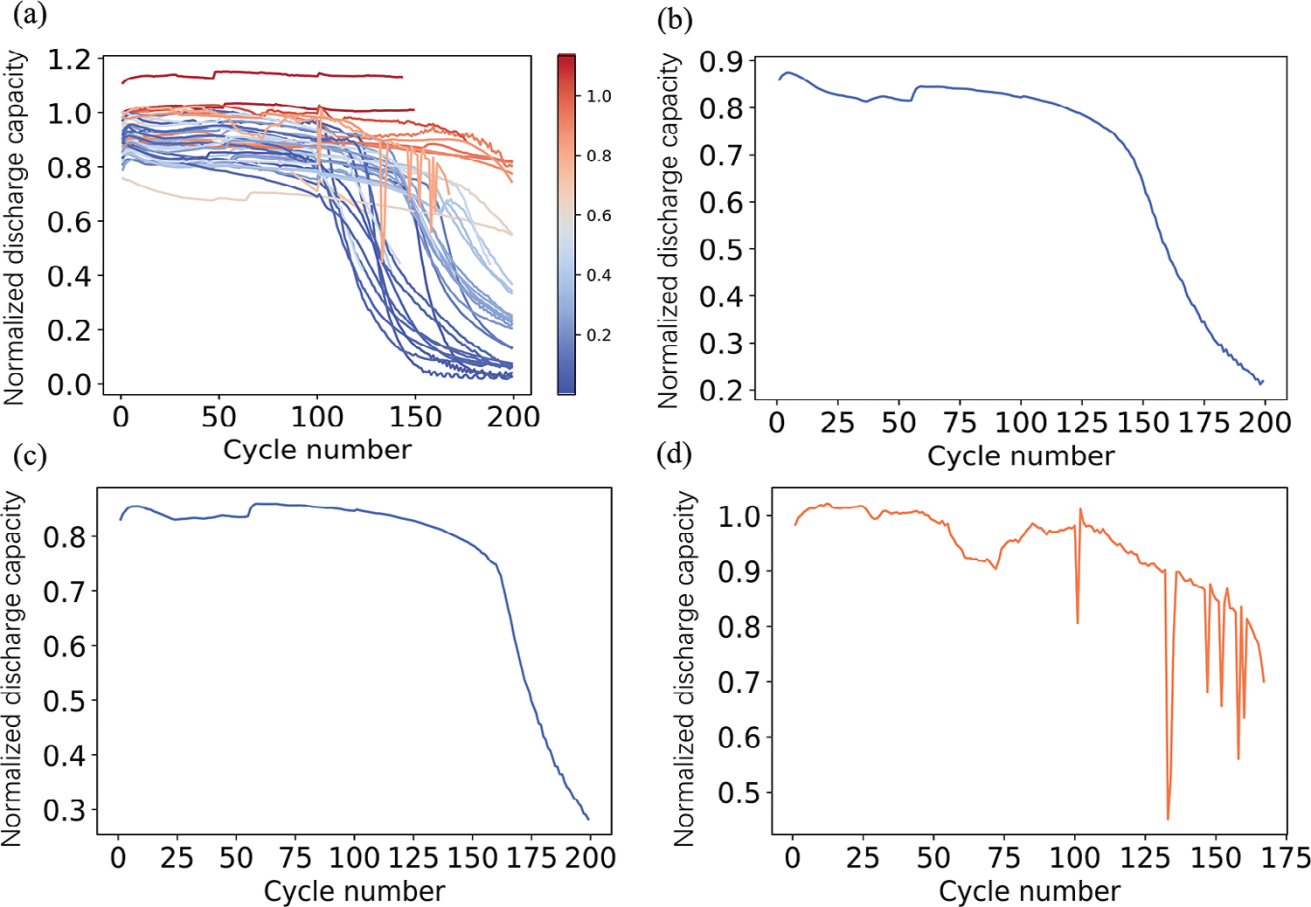
a) The discharge capacity degradation curves of the 48 examined cells. The color of each line is distinguished by the discharge capacity of the last cycle. b) Discharge capacity degradation curve of cell No. 40. c) Discharge capacity degradation curve of cell No. 39. d) Discharge capacity degradation curve of cell No. 9 which exhibits fluctuations.

In the present study, we prepared the cells with different technological parameters (kinds of electrolyte and separator). Besides, we also change the evaluation condition parameters, such as current density, separator thickness, and confining pressure which refers to the pressure exerted on the battery cell components during the cycling test. The detailed technological parameters of the cells investigated were summarized in Table S1 (Supporting Information). Such differences result in the change of life span among the cells. In **Figure** **5** **a,** the cells’ degradation trajectories of all investigated 48 cells as a function of cycle life was shown. In addition, the distribution of the lifespan of the cells is also shown in Figure S1 (Supporting Information). The end‐of‐life capacity is defined as when the discharge capacity falls to 80% of the nominal capacity (available maximum capacity).

Figure 5b shows the representative discharge profile of one cell (cell No. 40). The cell exhibited the 1st discharge capacity of 0.86 mAh with average discharge voltage of 3.7 V. With progress of cycle, the cell exhibited a capacity higher than 0.68 mAh up to the 138th cycle. After that the discharge capacity gradually decreased and reached to 0.21 mAh at 200th cycle. In Figure 5c, the profile of the different LMB cell with the same technological parameters (cell No. 39) was also shown. As the two profiles showed almost identical degradation trajectories, suggesting the high reproducibility of the cells investigated in the present study. Although most of cells investigated in the present study showed a similar capacity profile with that of cell No. 40, the unstable capacity profile was also observed among the cells. For example, in case of cell No. 9, there can be seen the fluctuation of capacity value after the 100th cycle (Figure 5d). Such unstable profile originated in the non‐uniform reaction characteristics of lithium metal electrode, such as, micro‐short or electrolyte shortage. Actually, after the 100th cycle, the over‐charging occurs, which is a typical deterioration mechanism of LMB. Cells of this type pose challenges in understanding their capacity degradation mechanism, which is both elusive and distinct from real‐world conditions. Consequently, we opted to eliminate certain cells from our dataset. Following our analysis, we have excluded eight cells from our project, leaving us with a total of 40 cells.

**Figure 6:**
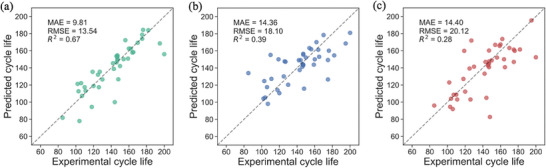
Parity plots for observed and predicted cycle lives for different feature sets using the ElasticNet model. a) discharge‐related feature subset. b) charge‐related feature subset. c) relaxation‐related feature subset.

Next, we applied discharge‐related features, charge‐related features, and relaxation‐related features independently to predict the cycle life using ElasticNet. We try to determine which feature subset could provide better prediction results. In Figure 6, from left to right it displays the prediction results for different feature subsets.

The *X* and *Y* axes in **Figure** **6** indicate the experimentally observed cycle life and mean value of ML predicted cycle life after the four‐fold cross‐validation respectively. Figure 6a shows the prediction result of using discharge‐related features, the testing MAE equals to 9.81, RMSE equals to 13.54 and the *R* ^2^ is 0.67, which is the best prediction performance among the three machine‐learning methods. For charge‐related features (Figure 6b) and relaxation‐related features (Figure 6c), the parity plots become more and more scattered, their MAE and RMSE become larger, meanwhile, the *R* ^2^ becomes smaller, meaning that the prediction results become worse than the first situation. However, despite their superiority, the prediction performance achieved using discharge‐related features alone, with an *R* ^2^ value of 0.67, did not meet our anticipated standards. Hence, to improve the prediction accuracy, we sought to optimize both the feature selection process and the machine learning methodology employed.

**Figure 7:**
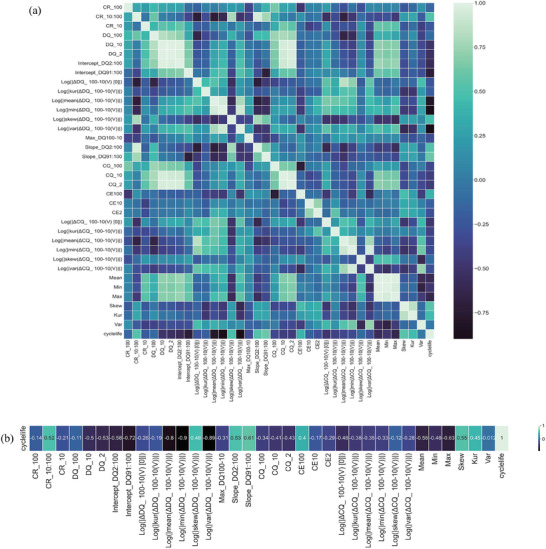
a) The relationship heatmap matrix of the variables in our dataset. b) Pearson's correlation coefficient of the features to the target value observed cycle lives of the 40 cells.

For the feature part, we mapped the Pearson's correlation coefficient heatmap matrix among variables in our data set including feature to feature and feature to target value which is shown in **Figure** **7**a.

It is a square matrix where rows and columns represent features and observed cycle life, and each cell contains the correlation coefficient between corresponding variables. The correlation coefficient between two features measures the magnitude and direction of the linear connection between those features, offering insights into how changes in one feature correspond to variations in another feature. The most important correlation in our research should focus on the relationship between the features and the observed cycle life which is the rightmost column in the correlation matrix. From the coefficient value we can attain a distinct comprehension of the correlation existing between them. Features with highe coefficient are regarded as important predictors to the cycle life.

From Figure 7b, the coefficients of discharge‐related features are relatively higher than those of charge‐related features and relaxation‐related features. Some of the features show strong correlation with the observed cycle life, for example, the most correlated feature is the logarithm of the minimum value of the Δ*DQ*_ 100–10_(V), which has a negative Pearson's correlation coefficient (*r* = −0.9) with the observed cycle life, next is the logarithm of the variance value of the Δ*DQ*_ 100–10_(V). However, there are several features that show a very weak correlation to the observed cycle life such as the discharge capacity of the 100th cycle, coulombic efficiency at the 100th cycle, and variance of the relaxation terminal voltage.

**Figure 8:**
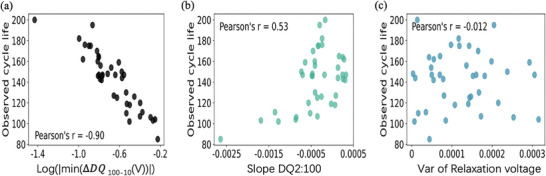
The cycle life of the cells plotted as a function for different features. a) cycle life as a function of the minimum value of the Δ*DQ*_100 − 10_(V). b) cycle life as a function of the slope of discharge capacity between the 2nd cycle and the 100th cycle. c. cycle life as function of the variance of the relaxation voltage between the 1st cycle to the 100t cycle.

Here, we choose several features of the cells in our dataset and plot the observed cycle life of the cells as a function of these features in **Figure** **8**.

In Figure 8a, the feature “log(|min(Δ*DQ*_ 100–10_(V))|)” exhibits a strong correlation as evidenced by their Pearson's correlation coefficient absolute value surpassing |0.8|. The cycle life of the cells has an obvious linear relationship with the feature. It's important to highlight that our calculations did not merely entail extracting the summary statistics of Δ*DQ*_ 100–10_(V); rather, we calculated the logarithm to the base 10 of these values. In our investigation, when addressing the logarithmic function, a Δ*DQ*_ 100–10_(V) approaching zero signifies a reduced disparity between the discharge capacity‐voltage curves, leading to more negative logarithmic values. According to the visual representation, the greater the negativity of the logarithmic value, the longer the observed cycle life of the cell—implying less noticeable capacity degradation.

Figure 8b illustrates the correlation between the slope of discharge capacity from the 2nd cycle to the 100th and cycle life, revealing a moderate correlation (*r* = 0.53). This plot demonstrates a gradual but not noticeable upward trend. For cells with shorter lifespans, the degradation tends to decline relatively faster, indicating a steeper slope, as the graph corroborates. In the case of cells with longer cycle life, their slopes are less negative, and the degradation trajectory exhibits a milder decline. However, by analyzing our dataset, we identified some uncertainties regarding cells with longer cycle life, which can be explained by the moderate correlation of this feature with cycle life.

In Figure 8c, the data points are scattered much more than the previous two features which signifies a weak correlation between observed cycle life and the variance of relaxation voltage from the 1st cycle to the 100th cycle. Therefore, this particular feature is considered as a poor predictor for cell cycle life. Based on our examination, we have identified nine discharge‐related features and three relaxation‐related features exhibiting strong or moderate correlation with cell cycle life. Nevertheless, all charge‐related features exhibit a weak correlation to cell cycle life. These 12 features are selected to use in the following research.

**Figure 9:**
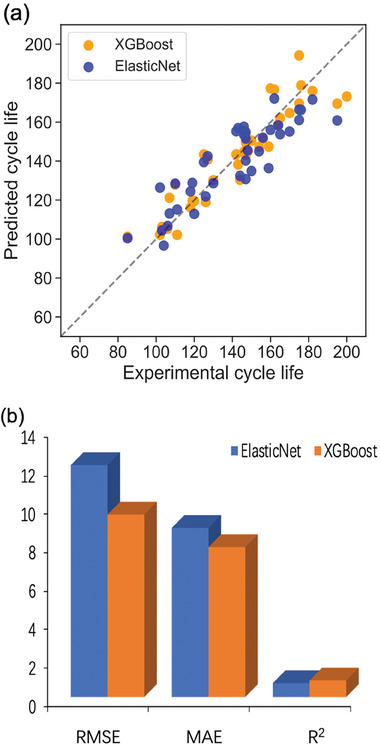
a). Parity plots of using ElasticNet and XGBoost on the 12 features. b). Prediction result histogram. The blue bar represents the results of ElasticNet and the Orange bar indicates XGBoost.

Next, for the ML method part, to leverage the predictive performance, we implemented the ElasticNet and XGBoost ML algorithms to the 12 selected features separately. The prediction results based on XGBoost and ElasticNet using the 12 selected features are shown in the **Figure** **9**.

From the parity plots, it's obvious the plot of ElasticNet is more scattered than that of XGBoost. In addition, based on the prediction results, it can be observed that XGBoost shows a slight decrease in RMSE and MAE, with values of 9.49 and 7.8, respectively, when compared to ElasticNet, where the RMSE and MAE are 12.06 and 8.8. Meanwhile, the *R* ^2^ of these two models are 0.86 and 0.72, which demonstrates non‐linear ML model XGBoost has a better prediction performance on the cell cycle life than ElasticNet. Hence, we decided to build our ML model based on XGBoost.

To eliminate the potential feature overfitting, we conducted exhaustive feature selection (EFS) on the 12 features. EFS is an approach that impartially evaluates the optimal feature subset using a specified evaluation metric. This method guarantees the assessment of all possible combinations, ensuring a comprehensive analysis without undue computational cost. Through EFS, a total of 4095 feature combinations were generated from the 12 features, each of the combinations was assessed by XGBoost with four‐fold cross‐validation, the optimal predictive performance for various feature numbers (*n*) was identified, and the outcomes are presented in **Table** **2**.

**Table 2:** The best *R* ^2^ score and the feature subsets when using exhaustive feature selection as a function of the feature numbers.

n	Best *R* ^2^	Feature combination
1	0.722	Log(|min(Δ*DQ*_ 100–10_(V))|)
2	0.854	Log(|min(Δ*DQ*_ 100–10_(V))|), slope_DQ2:100
3	0.871	Intercept_DQ91:100, Log(|min(Δ*DQ*_ 100–10_(V))|), Log(|var(Δ*DQ*_ 100–10_(V))|)
4	0.874	Intercept_DQ91:100, Log(|min(Δ*DQ*_ 100–10_(V))|), Log(|var(Δ*DQ*_ 100–10_(V))|), Slope_DQ91:100
5	0.884	Intercept_DQ91:100, Log(|min(Δ*DQ*_ 100–10_(V))|), Log(|var(Δ*DQ*_ 100–10_(V))|), Slope_DQ2:100, Mean
6	0.890	CR_10:100, Intercept_DQ91:100, Log(|min(Δ*DQ*_ 100–10_(V))|), Log(|var(Δ*DQ*_ 100–10_(V))|), Slope_DQ2:100, Mean
7	0.877	CR_10:100, Intercept_DQ91:100, Log(|min(Δ*DQ*_ 100–10_(V))|), Log(|var(Δ*DQ*_ 100–10_(V))|), Slope_DQ2:100, Slope_DQ91:100, Mean
8	0.885	CR_10:100, Intercept_DQ91:100, Log(|min(Δ*DQ*_ 100–10_(V))|), Log(|var(Δ*DQ*_ 100–10_(V))|), Log(|mean(Δ*DQ*_ 100–10_(V))|), Slope_DQ2:100, Mean, Slope_DQ91:100
9	0.871	CR_10:100, DQ_2, Intercept_DQ2:100, Intercept_DQ91:100, Log(|min(Δ*DQ*_ 100–10_(V))|), Log(|var(Δ*DQ*_ 100–10_(V))|), Log(|mean(Δ*DQ*_ 100–10_(V))|), Slope_DQ91:100, Mean
10	0.870	CR_10:100, DQ_2, Intercept_DQ2:100, Intercept_DQ91:100, Log(|min(Δ*DQ*_ 100–10_(V))|), Log(|var(Δ*DQ*_ 100–10_(V))|), Log(|mean(Δ*DQ*_ 100–10_(V))|), Slope_DQ91:100, Mean, Max
11	0.857	CR_10:100, DQ_2, Intercept_DQ91:100, Log(|min(Δ*DQ*_ 100–10_(V))|), Log(|var(Δ*DQ*_ 100–10_(V))|), Log(|mean(Δ*DQ*_ 100–10_(V))|), Slope_DQ2:100, Slope_DQ91:100, Mean, Max, Skew
12	0.859	All features

**Figure 10:**
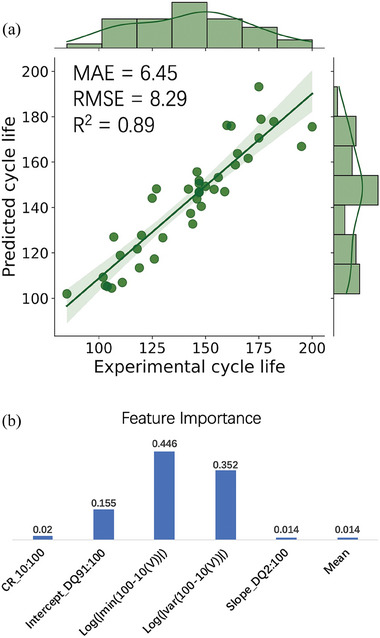
a) Parity plot of the best prediction using XGBoost with six features. b) Relative feature importance ranking for the six features in lifetime prediction.

Table 2 reveals that there is minimal variation in the scores within the range of *n* = 3 to *n *= 10. However, relatively large deviations are observed for values outside this range, both for *n* < 3 and *n* > 10. We find that the model provides the most accurate predictions with *n* = 6, resulting in an *R* ^2^ value of 0.89. The values of the six features of each cells are shown in the Table S2 (Supporting Information). The parity plot illustrating the use of this specific set of features is depicted in **Figure** **10**a in which the RMSE = 8.29 and MAE = 6.45.

According to this 6‐feature subset, we analyzed the feature importance. In Figure 10b, the feature importance of the six features is plotted, different feature has different relative importance to the model. In this feature subset, the discharge‐voltage‐related features such as the logarithm of the minimum value and the variance value of Δ*DQ*_ 100–10_(V) play crucial roles in the model performance, and the logarithm of the minimum value of Δ*DQ*_ 100–10_(V) (former) is the most important feature in this case. The slope of linear fit of the discharge capacity between the 2nd cycle and the 100th cycle, and the Mean value of the relaxation voltage have the same contribution to the model but their relative importance is lowest, the feature importance of capacity retention from 10 to 100 is higher than both of them, however, according to the Pearson's correlation coefficient, it has a weaker correlation to the observed cycle life compared with these two features, which indicates it has a higher ability to reduce the error of the prediction result, but it has less linear relationship with the cycle life. Through exhaustive feature selection method and use of advanced ML method XGBoost, we realized a satisfying ML model for LMB cycle life, which we name as “XGB‐LMBCLpredictor”.

**Figure 11:**
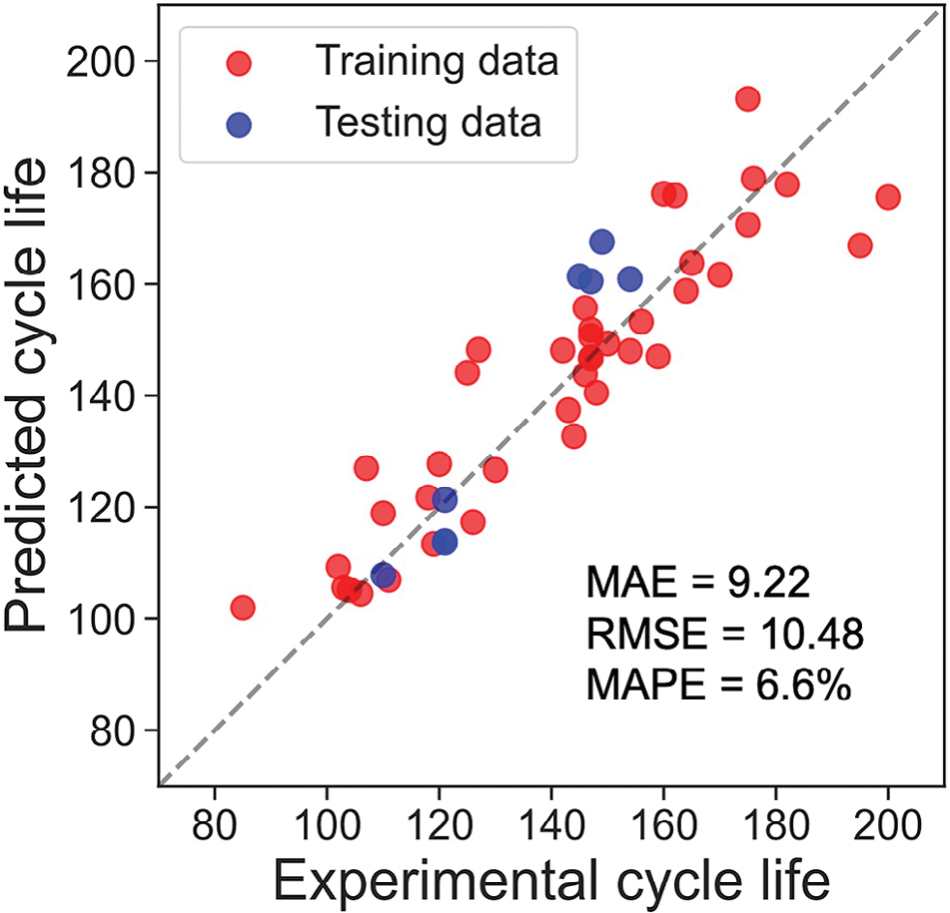
Prediction results of the eight new NMC811/Li metal cells, the red points indicate the training data, and the blue points represent the testing data.

Finally, we applied our model “XGB‐LMBCLpredictor” with eight new NMC811/Li metal cells as unseen data to test whether our model can predict the cycle life accurately. The details of the cells are shown in Supporting Information. Here, one cell is discarded because of its unstable capacity profile. The prediction result is shown in **Figure** **11**, the MAE and RMSE of the testing data are relatively small and the test error (Mean Absolute Percentage Error: MAPE) equals to 6.6%. The achieved RMSE and MAE values suggest that the model provides predictions with reasonable accuracy. The relatively low MAPE reinforces the model's accuracy, especially considering the percentage‐wise deviation. As indicated by these metrics, the model's performance is well‐suited for accurate cycle life predictions.

## 4 Conclusion

Utilizing machine learning modeling holds great potential for the diagnosis and prediction of batteries. It provides possibilities in various aspects, including their development, manufacturing, and optimization. In the present study, we focus on using the ML method to model the complex degradation mechanisms and predict the cycle life of NMC811/Li metal batteries via different features generated from different cycle processes. 48 of NMC811/Li metal batteries’ degradation data are recorded and features generated from the data are classified into three groups including discharge‐related features, charge‐related features, and relaxation‐related features. Linear regression model ElasticNet was first used in different feature groups, however the prediction performance was unsatisfactory, then by Pearson's correlation coefficient analysis, we selected 12 features out of the 35 features which have a strong or moderate correlation with the cycle life and applied non‐linear regression model XGBoost to predict the cycle life of the cells. Compared with the result of ElasticNet, XGBoost is much superior for cell cycle life prediction with an RMSE of around 9.49 and MAE 7.8. Exhaustive feature selection results show that six features out of the 12 features can give the best prediction result which decreases the RMSE to 8.29, MAE to 6.45, and increase *R* ^2^ to 0.89, where Log(|min(Δ*DQ*_ 100–10_(V))|) is found the most important feature, contributing more than 44% for lifetime prediction. Finally, by testing the unseen data, our best model achieves a 6.6% test error, which indicates our machine learning model “XGB‐LMBCLpredictor” is suitable for the cycle life prediction of LMB.

Through our investigation, utilizing the capabilities of machine learning algorithms, our goal is to attain heightened precision and dependability in predicting the cycle life of LMB. By expanding the horizons of predictive precision, our study has the potential to give clues of LMB advancement and implementation. This could lead to transformative outcomes in the realm of energy storage, ensuring enhanced safety, greater efficiency, and extended longevity for energy storage solutions.

## Conflict of Interest

The authors declare no conflict of interest.

## Supporting information

Supporting Information

## Data Availability

The data that support the findings of this study are available from the corresponding author upon reasonable request.
