# Supplementary material for: Data‐Driven Cycle Life Prediction of Lithium Metal‐Based Rechargeable Battery Based on Discharge/Charge Capacity and Relaxation Features
Source: Adv Sci (Weinh). 2024 Jun 27;11(33):2402608. doi: 10.1002/advs.202402608 (PMC11633329; doi:10.1002/advs.202402608)
Supplement: Supplementary file 1 — Supporting Information [file ADVS-11-2402608-s001.docx]

**Supporting information**

Data-driven Cycle Life Prediction of Lithium Metal based Rechargeable Battery based on Discharge/Charge Capacity and Relaxation Features

*Qianli Si^a,b^, Shoichi Matsuda^b,c^, Youhei Yamaji^b^, Toshiyuki Momma^a^, Yoshitaka Tateyama^a,b,c,d^*

1. *Department of Nanoscience and Nanoengineering, Faculty of Science and Engineering, Waseda University.*
2. *Research Center for Energy and Environmental Materials (GREEN), National Institute for Materials Science (NIMS)*
3. *NIMS-SoftBank Advanced Technologies Development Center, National Institute for Materials Science (NIMS)*
4. *Laboratory for Chemistry and Life Science, Institute of Innovative Research, Tokyo Institute of Technology.*

**Introduction of machine learning (ML) methods**

ElasticNet method

For ElasticNet linear regression model, the relationship between the observed values and the predicted values of the cell cycle life is defined as the following equation:

$$\begin{aligned} {\overset{^}{y}}_{i}=w_{0}+\sum_{i=1}^{n} w_{i}x_{i}\#\left( 1 \right) \end{aligned}$$

The variables "$w_{0}$" and "$w_{i}$" are introduced to assign distinct weights or values to the features. $x_{i}$ indicate the values of features. ${\overset{^}{y}}_{i}$ represents the predicted cycle life of one specific cell. n denotes the numbers of features used in the regression model. ElasticNet linear regression model are chosen since its enhanced effectiveness in cases where there are strong correlations between features, which can mitigate the risk of overfitting. ElasticNet constitutes a fusion of LASSO regression and ridge regression techniques whose resulting loss function can be represented as:

$J\left( w \right)= \frac{1}{2n}\sum_{i=1}^{n} ({\overset{^}{y}}_{i}({\overset{{}}{x}}_{ij})-{\overset{}{y}}_{i})^{2}$ + $(r\lambda\sum_{j=1}^{p} |w_{j}|)$ + $\left( \frac{1-r}{2}\lambda\sum_{j=1}^{p} w_{j}^{2} \right) (2)$

Which is consisted of three parts, the first part represents the square error between the predicted cycle life ${\overset{^}{y}}_{i}$ and the observed cycle life ${\overset{}{y}}_{i}$. The second and third parts encompass the L1-penalty ($\sum_{j=1}^{p} |w_{j}|$) and the L2-penalty ($\sum_{j=1}^{p} w_{j}^{2}$), respectively. The L1-penalty introduces the absolute value of the weight as a penalization term. Meanwhile, the L2-penalty employs the squared weight values. Combining the L1-penalty and the L2-penalty necessitates an additional parameter "r" alongside a hyperparameter "λ". In the context of machine learning, a hyperparameter is defined as a parameter that governs the learning process.

XGBoost method

The XGBoost technique is an adaptable and efficient tree-boosting system designed for scalability, flexibility, and portability. It applies machine learning algorithms within the Gradient Boosting framework. In contrast to multiple linear regression, XGBoost excels at managing non-linear associations. The mathematical representation of the tree, denoted as f(x), is as follows:

$$\begin{aligned} f_{t}\left( x \right)=\omega_{q\left( x \right)},\left( q:R^{d}\to\left\{ 1,2,\ldots,T \right\},\omega\in R^{T} \right)\#\left( 3 \right) \end{aligned}$$

where 't' denotes a tree, 'q' represents the configuration of each tree that links an instance to its respective leaf position, and 'T' indicates the total number of leaves within the tree. Each 'ft' corresponds to a distinct tree configuration 'q' and leaf values 'ω' (the outcome of a tree).

The objective function is:

$obj^{(t)}=\sum_{i=1}^{n} l(y_{i},{\overset{^}{y}}_{i}^{(t)})+\sum_{i=1}^{t} \Omega(f_{i})$ (4)

'𝑙' stands for a continuously differentiable convex loss function that quantifies the disparity between the prediction '$\overset{^}{y}$' and the target '$y_{i}$'. The second component 'Ω' imposes a penalty on the intricacy of the model, aiding in the regularization of the ultimately learned weights to prevent overfitting.

$$\begin{aligned} \Omega\left( f \right)=\gamma T+\frac{1}{2}\lambda\sum_{j=1}^{T} \omega_{j}^{2}\#\left( 5 \right) \end{aligned}$$

Here, 'ωj' represents the weight assigned to the 'j-th' leaf node. 'γ' and 'λ' are the coefficients that determine the strength of the penalty term 'Ω'.

$$obj^{(t)}=\sum_{i=1}^{n} l\left( y_{i}{\overset{{}}{\overset{^}{,y}}}_{i}^{\left( t-1 \right)}+f_{t}\left( x_{i} \right) \right)+\Omega\left( f_{t} \right)+constant$$

$$\begin{aligned} \approx\sum_{i=1}^{n} \left( l\left( y_{i}{\overset{{}}{\overset{^}{,y}}}_{i}^{\left( t-1 \right)} \right)+g_{i}f_{t}\left( x_{i} \right)+\frac{1}{2}h_{i}f_{t}^{2}\left( x_{i} \right) \right)+\Omega\left( f_{t} \right)+constant\#\left( 6 \right) \end{aligned}$$

Here,$g_{i}=\partial_{{\overset{^}{y}}^{(t-1)}}l(y_{i},{\overset{^}{y}}^{(t-1)})\text{ and }h_{i}=\partial_{{\overset{^}{y}}^{(t-1)}}^{2}l(y_{i},{\overset{^}{y}}^{(t-1)})$, the objective function change to the following equation after remove constant

$$\begin{aligned} obj^{\left( t \right)}d=\sum_{i=1}^{\left( t \right)} \left( g_{i}\omega_{q\left( x_{i} \right)}+\frac{1}{2}h_{i}\omega_{q\left( x_{i} \right)}^{2} \right)+\gamma T+\frac{1}{2}\lambda\sum_{j=1}^{T} \omega_{j}^{2}\#\left( 7 \right) \end{aligned}$$

$$\begin{aligned} =\sum_{j=1}^{T} \left( G_{j}\omega_{j}+\frac{1}{2}\left( H_{j}+\lambda\right)\omega_{j}^{2} \right)+\gamma T\#\left( 8 \right) \end{aligned}$$

Where $G_{j}= \sum{i\in I_{j}g}_{i},H_{j}=\sum{i\in I_{j}h}_{i},I_{j}=\left\{ i | q\left( x_{i}=j \right) \right\}.$

The optimal weight '$\omega_{j}^{*}$' for the 'j-th' leaf node within a given structure 'q(x)' can be calculated as follows:

$$\begin{aligned} \omega_{j}^{*}=-\frac{G_{j}}{H_{j}+\lambda}\#\left( 9 \right) \end{aligned}$$

The optimal loss is:

$$\begin{aligned} obj^{*}=-\frac{1}{2}\sum_{j=1}^{T} \frac{(G_{j})^{2}}{H_{j}+\lambda}+\gamma T\#\left( 10 \right) \end{aligned}$$

The function '$obj^{*}$' is a measure of the quality of a tree structure and is dependent on the characteristics of the tree being evaluated. A lower value of '$obj^{*}$' indicates a better or more favorable tree structure.

To prevent overfitting, both of the hyperparameters of the ElasticNet and XGBoost machine learning methods are set by using 5-fold cross-validation procedure. For each fold, four subsets are utilized for training the model, while the remaining subset is reserved for validation. The model is trained and evaluated five times, with each subset serving as the validation set once. This iterative process enables a comprehensive assessment of the model's performance across different hyperparameter configurations, thereby facilitating the selection of optimal hyperparameters that generalize well to unseen data.

**Definition of the features**

For the 17 discharge features:

Six features were calculated as the summary statics including minimum, variance, skewness, kurtosis, mean and first value of the$\Delta DQ$(V) from the discharge capacity-voltage curves including Log(|min($\Delta DQ$_100-10_(V))|),Log(|mean($\Delta DQ$_100-10_(V))|), Log(|var($\Delta DQ$_100-10_(V))|),Log(|$\Delta DQ$_100-10_(V) [0]|), Log(|skew($\Delta DQ$_100-10_(V))|), Log(|Kur($\Delta DQ$_100-10_(V))|), the logarithm value are calculated to normalize those data. 3 features are Discharge capacity of 2, 10, and 100, which means the exact discharge capacity of two, ten, and one hundred cycle. In addition, The Slope and Intercept of the linear fit to the capacity fade curve, cycles 2 to 100, 91 to 100, these features are generated by calculating the slope of the discharge capacity of 2 to 100, and 91 to 100 (connect two points to get one line) by using the discharge capacity / cycle life curve. The intercept features are extracted by the intercept of each line on the Y-axis. Max discharge capacity difference of 100 and 2 cycle is using the discharge capacity of 100 to minus it of 2 cycle. The capacity retention _1:100, 2:1, 99:100 are calculated by $CR=\frac{C_{Dch\left( n \right)}}{C_{Dch\left( n-1 \right)}}$.

For the 12 charge features:

Six features were calculated as the summary statics including minimum, variance, skewness, kurtosis, mean and first value of the$\Delta CQ$(V) from the discharge capacity-voltage curves including Log(|min($\Delta CQ$_100-10_(V))|),Log(|mean($\Delta CQ$_100-10_(V))|), Log(|var($\Delta CQ$_100-10_(V))|),Log(|$\Delta CQ$_100-10_(V) [0]|), Log(|skew($\Delta CQ$_100-10_(V))|), Log(|Kur($\Delta CQ$_100-10_(V))|). And also Charge capacity of 2, 10, and 100 which is similar to discharge related features. For the coulombic efficiency of 2, 10, and 100 which are calculated by $CE=\frac{C_{Dch\left( n \right)}}{C_{Ch\left( n \right)}}$.

For the 6 relaxation features:

Including minimum, maximum, variance, skewness, kurtosis, and mean of the terminal voltage from the 1 cycle to the 100 cycle from the relaxation voltage-time curves. Here, Skewness measures the asymmetry of the distribution of values in a dataset. Kurtosis measures the peakedness or flatness of the distribution of values in a dataset.

**Supporting Tables**

Technological parameters of the Li-metal rechargeable batteries (LMB)

| ML construction data | | | | | |
| --- | --- | --- | --- | --- | --- |
| Cell ID | Electrolyte | Separator thickness | Confining pressure | Discharge current density | Charge current density |
|  |  |  | kPa | mA/cm2 | mA/cm2 |
| 1 | 4M_LiFSI in DME | 18 | 100 | 6 | 0.6 |
| 2 | 4M_LiFSI in DME | 18 | 100 | 6 | 0.6 |
| 3 | 4M_LiFSI in DME | 18 | 100 | 0.6 | 3 |
| 4 | 4M_LiFSI in DME | 18 | 100 | 0.6 | 3 |
| 5 | 4M_LiFSI in DME | 18 | 300 | 6 | 0.6 |
| 6 | 4M_LiFSI in DME | 18 | 300 | 6 | 0.6 |
| 7 | 4M_LiFSI in DME | 18 | 300 | 0.6 | 3 |
| 8 | 4M_LiFSI in DME | 18 | 300 | 0.6 | 3 |
| 9 | 4M_LiFSI in DME | 18 | 600 | 6 | 0.6 |
| 10 | 4M_LiFSI in DME | 18 | 600 | 6 | 0.6 |
| 11 | 4M_LiFSI in DME | 18 | 600 | 0.6 | 3 |
| 12 | 4M_LiFSI in DME | 18 | 600 | 0.6 | 3 |
| 13 | 1M_LiPF6 in FEC/EMC(1:3vol%) | 18 | 100 | 6 | 0.6 |
| 14 | 1M_LiPF6 in FEC/EMC(1:3vol%) | 18 | 100 | 6 | 0.6 |
| 15 | 1M_LiPF6 in FEC/EMC(1:3vol%) | 20 | 100 | 6 | 0.6 |
| 16 | 1M_LiPF6 in FEC/EMC(1:3vol%) | 20 | 100 | 6 | 0.6 |
| 17 | 0.6 M LiDFOB and 0.6 M LiBF4 in FEC:DEC (1:2 v:v) | 18 | 100 | 6 | 0.6 |
| 18 | 0.6 M LiDFOB and 0.6 M LiBF4 in FEC:DEC (1:2 v:v) | 18 | 100 | 6 | 0.6 |
| 19 | 4M_LiFSI in DME | 18 | 100 | 6 | 3 |
| 20 | 4M_LiFSI in DME | 18 | 100 | 6 | 3 |
| 21 | 4M_LiFSI in DME | 20 | 100 | 6 | 3 |
| 22 | 4M_LiFSI in DME | 20 | 100 | 6 | 3 |
| 23 | 4M_LiFSI in DME | 32 | 100 | 6 | 3 |
| 24 | 4M_LiFSI in DME | 32 | 100 | 6 | 3 |
| 25 | 4M_LiFSI in DME | 14 | 100 | 6 | 3 |
| 26 | 4M_LiFSI in DME | 14 | 100 | 6 | 3 |
| 27 | 4M_LiFSI in DME | 10 | 100 | 6 | 3 |
| 28 | 4M_LiFSI in DME | 10 | 100 | 6 | 3 |
| 29 | 4M_LiFSI in DME | 27 | 100 | 6 | 3 |
| 30 | 4M_LiFSI in DME | 27 | 100 | 6 | 3 |
| 31 | 4M_LiFSI in DME | 19 | 100 | 6 | 3 |
| 32 | 4M_LiFSI in DME | 19 | 100 | 6 | 3 |
| 33 | 4M_LiFSI in DME | 23 | 100 | 6 | 3 |
| 34 | 4M_LiFSI in DME | 23 | 100 | 6 | 3 |
| 35 | 4M_LiFSI in DME | 15 | 100 | 6 | 3 |
| 36 | 4M_LiFSI in DME | 15 | 100 | 6 | 3 |
| 37 | 4M_LiFSI in DME | 11 | 100 | 6 | 3 |
| 38 | 4M_LiFSI in DME | 11 | 100 | 6 | 3 |
| 39 | 4M_LiFSI in DME | 10 | 100 | 6 | 3 |
| 40 | 4M_LiFSI in DME | 10 | 100 | 6 | 3 |
| 41 | 4M_LiFSI in DME | 22 | 100 | 6 | 3 |
| 42 | 4M_LiFSI in DME | 22 | 100 | 6 | 3 |
| 43 | 4M_LiFSI in DME | 23 | 100 | 6 | 3 |
| 44 | 4M_LiFSI in DME | 23 | 100 | 6 | 3 |
| 45 | 4M_LiFSI in DME | 13 | 100 | 6 | 3 |
| 46 | 4M_LiFSI in DME | 13 | 100 | 6 | 3 |
| 47 | 4M_LiFSI in DME | 19 | 100 | 6 | 3 |
| 48 | 4M_LiFSI in DME | 19 | 100 | 6 | 3 |
| Unseen data | | | | | |
| 49 | 4M_LiFSI in DME | 18 | 100 | 1.5 | 0.6 |
| 50 | 4M_LiFSI in DME | 18 | 100 | 1.5 | 0.6 |
| 51 | 4M_LiFSI in DME | 18 | 100 | 1.5 | 0.6 |
| 52 | 4M_LiFSI in DME | 18 | 100 | 3 | 3 |
| 53 | 4M_LiFSI in DME | 18 | 100 | 3 | 3 |
| 54 | 4M_LiFSI in DME | 18 | 100 | 3 | 3 |
| 55 | 4M_LiFSI in DME | 18 | 100 | 6 | 3 |
| 56 | 4M_LiFSI in DME | 18 | 100 | 6 | 3 |
| 57 | 4M_LiFSI in DME | 18 | 100 | 6 | 3 |

**Table S1.** The experimental dataset of 57 cells. The data of the first 48 cells were used to construct the ML models. The last 9 cells were used as unseen data to test our model’s performance.

Values of the 6 selected features

| **CR_10:100** | **Intercept_DQ91:100** | **Log(\|min(**$\boldsymbol{\Delta}\boldsymbol{D}\boldsymbol{Q}$**_100-10_(V))\|)** | **Log(\|var(**$\boldsymbol{\Delta}\boldsymbol{D}\boldsymbol{Q}$**_100-10_(V))\|)** | **Slope_DQ2:100** | **Mean** |
| --- | --- | --- | --- | --- | --- |
| 0.84621037 | 0.97261773 | -0.3763331 | -1.8653635 | -0.0013568 | 3.32333847 |
| 0.95148274 | 0.96215127 | -0.9000396 | -3.1706152 | -0.0003345 | 3.23506804 |
| 0.90758807 | 0.92363115 | -0.5783613 | -2.398176 | -0.0007303 | 3.2024267 |
| 0.73758545 | 1.26205407 | -0.2258565 | -1.6830454 | -0.0026458 | 3.33081508 |
| 0.93689524 | 1.0276355 | -0.8106463 | -2.9040349 | -0.0004797 | 3.20256198 |
| 0.95284659 | 1.06938303 | -0.6247489 | -2.4727447 | -0.0003528 | 3.23315756 |
| 0.98754533 | 0.99171729 | -0.6560857 | -2.4007702 | 8.59E-05 | 3.21294009 |
| 1.00666539 | 0.89493529 | -0.9584541 | -2.9684639 | 0.00028069 | 3.23058216 |
| 0.94096102 | 1.03164854 | -0.7696147 | -2.8625538 | -0.0004687 | 3.21274246 |
| 0.97944708 | 0.90753359 | -0.6816565 | -2.5042307 | 0.0001366 | 3.23086345 |
| 0.98465554 | 0.88632966 | -0.399462 | -1.7930267 | -0.0003501 | 3.25821721 |
| 0.9947079 | 1.05448379 | -0.7902738 | -2.6847906 | 0.0002542 | 3.23319275 |
| 0.9927814 | 0.93212545 | -0.8032492 | -2.7362397 | 0.00024608 | 3.24515864 |
| 0.91721365 | 0.86593504 | -0.5568613 | -2.3410926 | -0.0007038 | 3.22842475 |
| 0.94475513 | 0.90094986 | -0.3973999 | -1.8915949 | -0.0005608 | 3.261795 |
| 0.99843056 | 1.04598191 | -0.7759624 | -2.6305323 | 0.00030594 | 3.25322952 |
| 0.97501715 | 1.14437634 | -0.500586 | -2.0234137 | 6.57E-05 | 3.24507649 |
| 0.95133025 | 0.93295343 | -0.6419883 | -2.4670448 | -0.0001827 | 3.2216029 |
| 0.98843169 | 0.8313599 | -1.4319403 | -3.1349561 | -0.0001663 | 3.25620762 |
| 0.88826917 | 1.13476787 | -0.252318 | -1.5799072 | -0.0008657 | 3.24491222 |
| 0.87580789 | 1.07597452 | -0.2965553 | -1.7379619 | -0.0008519 | 3.24359366 |
| 0.85881189 | 1.23653617 | -0.5028543 | -2.4146717 | -0.0012751 | 3.34269719 |
| 1.00089539 | 0.99401486 | -0.8019251 | -2.6757741 | 0.00032362 | 3.26079322 |
| 0.9415802 | 0.78056511 | -0.8643708 | -3.0060613 | -0.0002985 | 3.18353236 |
| 0.93855415 | 1.14290744 | -0.5284112 | -2.206069 | -2.62E-05 | 3.24327334 |
| 0.9668629 | 0.99458986 | -0.4554142 | -1.9915023 | -0.0002552 | 3.26461132 |
| 0.91772707 | 0.85410896 | -0.607687 | -2.4557474 | -0.0006407 | 3.2079805 |
| 0.94509288 | 0.834534 | -0.8138406 | -2.8959062 | -0.000414 | 3.20930677 |
| 0.802861 | 0.9806727 | -0.27266 | -1.5702598 | -0.0016898 | 3.30783656 |
| 0.9939148 | 0.96675866 | -0.7669021 | -2.6275458 | 0.00024291 | 3.24028626 |
| 0.95629589 | 0.83299398 | -0.8832529 | -3.0996928 | -0.0003288 | 3.21869122 |
| 1.00002822 | 1.16806556 | -0.5758456 | -2.2351623 | 0.00013585 | 3.32770774 |
| 0.96786833 | 0.85748363 | -0.9390991 | -3.2070313 | -0.0002415 | 3.2212857 |
| 0.96258016 | 0.91133798 | -0.5354564 | -2.1752479 | -0.000407 | 3.24425343 |
| 0.96398312 | 0.8410738 | -0.9959647 | -3.3541782 | -0.0002403 | 3.21692229 |
| 0.94094989 | 0.87214626 | -0.8201049 | -2.9855995 | -0.0004539 | 3.21080963 |
| 0.99509221 | 1.0540217 | -0.5332367 | -2.1420546 | -0.0005228 | 3.32855269 |
| 0.96406448 | 1.06064328 | -0.7164 | -2.6253774 | -0.0004696 | 3.33011696 |
| 0.93982472 | 0.91817072 | -0.7861158 | -2.8692112 | -0.0004095 | 3.20295322 |
| 0.99134659 | 0.91247499 | -0.8055679 | -2.7265036 | 0.00016064 | 3.24773666 |

**Table S2.** The values of the 6 selected features which give the best prediction result for the 40 cells, finally used to construct the ML model.

**Supporting Figure**


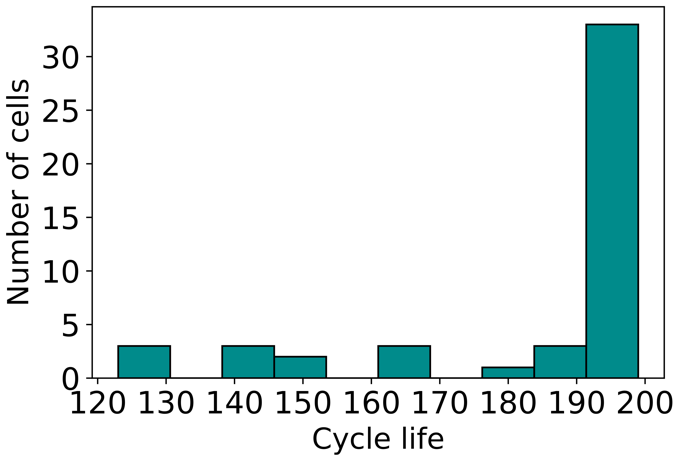


**Figure S1.** The distribution of the cycle life of the 48 examined cells.
